# Supplementary material for: Comparison of Azathioprine-Induced Pancreatitis and Gastrointestinal Intolerance in IBD: Role of Demographics, Clinical Variables, and HLA DQA1/DRB1 Alleles
Source: J Clin Med. 2025 Dec 2;14(23):8539. doi: 10.3390/jcm14238539 (PMC12692811; doi:10.3390/jcm14238539)
Supplement: Supplementary file 1 [file jcm-14-08539-s001.zip › jcm-3968477-supplementary.pdf]

Supplementary Table S1. Comparison of AZA Treatment Characteristics Across Study Groups

	Control Group (n=88)	AZA-AP Group (n=44)	GI-INT Group (n=44)
**Overall duration of AZA treatment**	70 (85.8), mo	2-424 (4), week	1.2 ± 1, day
**AZA dose at AP/GI-INT onset, mean ± SD (mg/day)**	Not applicable	95 ± 35.99*	57.38 ± 19.12 *

AZA: azathioprine; * AZA dose at the time of event between AZA-AP and AZA-GI-INT groups was not significantly different, and for all patients aimed AZA starting dose was 50 mg/day. AZA-GI-INT group's (within less than 3 days after initiation of AZA) mean AZA level, at the time of event, higher than 50 mg comes from 6 patients, whose initial AZA starting doses were higher than 50 mg/day.
